# Dyad placement in general practice: interview study with Norwegian medical students and their clinical supervisors

**DOI:** 10.1186/s12909-025-08405-y

**Published:** 2025-12-11

**Authors:** Julie Solberg Knutsen, Steinar Hunskaar, Gunnar Tschudi Bondevik, May-Lill Johansen

**Affiliations:** 1 Department of Global Public Health and Primary Care, University of Bergen, Bergen, Norway; 2 National Centre for Emergency Primary Health Care, NORCE Research AS, Bergen, Norway; 3 General Practice Research Unit, Department for Community Medicine, UiT The Arctic University of Norway, Tromsø, Norway

## Abstract

**Background:**

Clinical placement for medical students in general practice is a key component of the primary healthcare curriculum and has proven to be a strong recruitment arena for general practice. Because of supervisor shortages and an increasing number of medical students, placing two students together with one supervisor is a potential option. While such dyad placement has been explored in undergraduate skills and simulation training, there is less research from teaching medical students in general practice. If a lack of capacity forces universities into new placement structures, it is important to evaluate the benefits and challenges in general practice settings. This study therefore aimed to investigate dyad placements in general practice.

**Methods:**

We performed a qualitative study in which eight 6^th^-year medical students and three clinical supervisors (general practitioners, GPs) were interviewed individually or in groups. All participants were involved in dyad placements at the University of Bergen, Norway, during spring 2023. Audio recordings from interviews were transcribed verbatim and analysed thematically using systematic text condensation. The theory of psychological safety was used to frame the discussion.

**Results:**

Dyad placements were organised in multiple ways according to preferences, needs and prerequisites, showing that there are many ways to make such placements work. Supervisors experienced feasibility despite their initial concerns about workload and time to follow up each student. They also illustrated situations where dyad placement could be challenging. Students saw many patients and assessed dyad placement as not to be a hindrance to this. The student dyad provided a safe space, resulting in rich learning experiences and enhanced social integration in the work environment at the surgery. Students learned from each other’s clinical strengths.

**Conclusions:**

Dyad placement added peer teaching and peer learning to students’ clinical experiences. By supporting each other’s presence and learning processes, the students experienced psychological safety, which is known to be a prerequisite for beneficial learning behaviour. We therefore argue that dyad placement can provide students with educational advantages. With a consulting room and sufficient patients available for the students, the supervisors assessed dyad placement as feasible in general practice.

## Background

Clinical placements are important learning arenas for medical students, ensuring that they gain real patient learning [1]. To obtain positive placement experiences, enough supervisors who are motivated to support and teach are needed. Due to rising numbers of medical students, several universities experience capacity problems regarding clinical placements [2]. So also for general practice, where an increasing workload [3] and a general practitioner (GP) shortage both affect supervisor availability [4, 5]. The lack of available student consulting rooms is a critical limitation in general practice [6, 7]. However, we recently reported that there still is sufficient capacity and willingness among Norwegian GPs to host an increasing number of medical students in clinical placements if specific prerequisites are fulfilled [6, 8].

Different strategies for recruiting more clinical teachers have been proposed, such as enhanced communication between educational teams and clinical teachers, regular practice visits, providing formal recognition of the supervisor role, and sufficient financial remuneration [9]. Additionally, the use of GP registrars as supervisors for medical students has been implemented in several countries [10]. However, the rapid increase in student numbers often exceeds the availability of supervisors.

Clinical placement in general practice is known for its apprenticeship model and one-to-one relationship between student and supervisor. However, to decrease the discrepancy between increasing student numbers and a lack of supervisors, two same-level learners can be placed with the same supervisor in a dyad placement [11, 12]. Peer-assisted learning (PAL), where students with no professional role as teachers help each other learn and thereby learn themselves, has proven valuable for learning practical and clinical skills [13]. A review of same-level PAL in clinical placements for medical students concluded that it contributes to the development of teaching, procedural and collaborative skills, evaluative judgement, and knowledge [14]. However, none of the included studies were from general practice placements. To our knowledge, the only study from a general practice placement setting is Danish and investigated medical students’ and GPs’ experiences with a ten-day dyad placement [12]. The authors concluded that dyad practice was both feasible and suitable in a general practice clinical setting. As structural changes can raise concerns, it is important to explore and communicate existing experiences with dyad placement in general practice.

The present study aimed to investigate last-year medical students’ and their supervisors’ experiences with six-week dyad placements in general practice in Norway.

## Design, materials and methods

### Context

At the University of Bergen, Norway, a dyad placement model was piloted for ten students during spring 2023 due to supervisor capacity challenges. During a six-week clinical placement in the final year, medical students are expected to learn the work of GPs in a supervised real clinical general practice setting. Both urban and rural general practice surgeries are used, and most students have to relocate for their placement period. The students choose the placement site from a list of available general practice surgeries. The students receive random numbers, and choose in turn, starting with number one. This means that student number one chooses from a full list of surgeries, while student number 89 only has a few options left. The students were able to deduce which placement sites offered dyad placement, based on number of students assigned to each surgery.

### Design

We employed a qualitative interview study design to explore the experiences of a six-week dyad placement from both the students’ and supervisors’ perspectives. By using an exploratory and qualitative approach, we aimed for a rich and detailed understanding of the participants’ experiences with the complex learning setting that can be found in clinical placements. In the discussion section, some of the results are interpreted in greater depth through the theory of psychological safety [15].

### Participants

Three GPs and ten medical students, comprising all participants in the dyad placement pilot, were invited to be interviewed. Two students were unable to attend. The GPs formed one focus group, while six students took part in individual interviews. A dyad of two students was interviewed together. The GPs were men aged 38–65 years and were all experienced supervisors working at places of variable rurality, ranging from a big city to a small city and a more rural town. The students included one male and seven females, all of whom were in their 6^th^ year of the undergraduate programme.

### Data collection

JSK conducted the semi-structured interviews lasting 60 to 90 min using a pre-developed interview guide (Table 1). Three interviews were digital (video), and five were in-person. MLJ joined the focus group discussion as a co-moderator. The audio recordings were transcribed verbatim and anonymised.

**Table 1:** Topics guiding the interviews

For supervisors (focus group)	For students
What made you say yes to host two students for this clinical placement?	Tell about a typical day in the placement. - How were you organised? - How did you interact?
How was it to supervise two students in the same placement period? - Benefits? - Challenges?	How was it to be in the role of a peer teacher and learner?
How would you assess your time to supervise two students instead of one?	How did you feel prepared for such a placement period?
How had dyad placement affected the environment on the workplace (the surgery)?	What have you learned from being together in this placement?
	To what extent is it essential to choose your peer for the placement?
How is clinical placement in general practice suited for dyad placements?	How is clinical placement in general practice suited for dyad placements?
Would you continue to offer dyad placements in your surgery? - Why?	If you were asked by a student in the class below whether to choose single or dyad placement, what would you recommend? - Why?

### Data analyses

We analysed the transcripts using Systematic text condensation (STC), a thematic, cross-case analysis for qualitative data [16]. STC consists of the following steps: (1) getting a total impression and identifying preliminary themes by reading all transcripts; (2) identifying, classifying and sorting meaning units related to the preliminary themes into code groups; (3) abstracting condensates from each code group and its subgroups; and (4) re-conceptualising condensates and quotations into analytical text with descriptions and category headings. The analysis was performed iteratively, going back and forth between the stages to deepen understanding, identify new insights and refine emerging themes. JSK and MLJ performed steps 1–3 together, while JSK performed step 4 independently. We completed steps 1–3 for three of the interviews and then included the other interviews in the analysis.

### Reflexivity

All researchers are medical doctors with most of their clinical and scientific experience in general practice and primary care. We are all involved in undergraduate teaching in general practice. All the authors are involved in developing high-quality clinical placements, while MLJ and GTB also have practical tasks related to them. In the author group we did not have unanimous preconceptions about dyad placement and its potential benefits and challenges for learning. This led to constructive and balanced discussions, along with extra caution in our interpretations.

### Ethical considerations

The study was approved by Sikt, the Norwegian Agency for Shared Services in Education and Research (ref. 224868). Participation was voluntary and did not influence the students’ assessments or the GPs’ formal roles as supervisors. All eleven participants agreed and gave their consent for their interviews to be audio-recorded and analysed for research purposes. The audio recordings were stored on a safe university server. Throughout the results section, all the students are referred to as female to maintain their anonymity.

## Results

The students’ and supervisors’ explanations and reflections on dyad placement included experiences of success and feasibility, yet they also illustrated situations where dyad placement could be challenging. Notably, there were many ways to make dyads work, with the key being the tailoring of organisational and pedagogical structures. Supervisors experienced that despite their initial concerns, the workload and time to follow up each student was adequate, given an available consulting room and patients. The students experienced satisfactory patient exposure relative to their expectations and initial concerns. They felt that the dyad was a safe space where they supported one another and learned from each other’s clinical strengths. Below, we elaborate on the students’ and supervisors’ experiences with dyad placement.

### Many ways to make dyad placements work

In a class of 90 students, some participants knew each other well, whereas others did not. Two students highlighted that their positive placement experience was a result of knowing each other in advance. Some of the students who did not know their peer spoke about great teamwork as colleagues. One student said:

“It does not have to be a good friend to feel safety in having the other student there. As long as we are not competing for anything.” (Student 3).

The students collaborated in patient consultations, observed each other, worked independently and engaged in a mix of these activities. One student, who had her own consulting room and worked mostly independently, believed that she learned more from working alone in the role of a doctor than from observing others. The students who collaborated a lot described the benefits of having a discussion partner before, during and after the consultation.

When you think out loud or discuss things, it tends to stick better. It’s somewhat like a colloquium in a way. (Student 8)

The three supervisors described three different preconditions related to surgery facilities. One of them had two student consulting rooms, the second had one, and the third GP had no dedicated student consulting room. The latter explained that the challenge of finding rooms made the workday quite stressful, and he would not recommend dyad placement in a surgery without a dedicated student consulting room. He also highlighted the importance of having enough patients available for the students as a prerequisite.

To administer the weeks, set up a plan, and feel that you give good quality for six weeks to two students instead of one (…) However, we might be in another situation than others. We do not have waiting lists and we struggle with enough consulting rooms. (Supervisor 1)

All students expressed satisfaction with their placement experience. Even though the dyads performed the placement differently, they all recommended dyad placement for their fellow students. However, many noted that their surgery’s way of organising the placement would be the preferred one.

I would recommend dyad placement with someone they know well (…) or at least a person you think you can function with every day. (Student 7)

I was called by a student in the class below me, wondering where to choose placement. I recommended her to organise, if possible, a dyad rather than being alone, because that would have felt lonelier and more stressful. (Student 6)

### Some concerns about dyad placement were refuted

The GPs expressed concerns about the individual follow-up of the students. In a dyad where the students differed in motivation during the placement, one GP thought that he could have given more attention to the less motivated student if she were alone. Nevertheless, he observed that the students encouraged and motivated each other throughout the placement period. Another GP experienced students with varying degrees of confidence and drive. He stated that the structure of the dyad consultations then was especially important. He proposed to alternate the role as doctor between the two students, thus ensuring equal challenges and educational outcomes.

One of them should sit in the background [as an observer] (…) and they must give each other feedback after each patient. We have to talk about that early in the process – how to give feedback. (Supervisor 1)

There is an increased workload for the supervisor with two students instead of one. However, one of the GPs found it a relief when students consulted patients together. They helped each other slightly further before conferring with the supervisor.

Initially we thought it could be more workload, but it ended up not being as much as we thought (…) The workload can increase when they become more independent, doubled up with questions, but overall, it is not twice as much work. (Supervisor 3)

The students expressed concerns that being in dyads would result in less clinical experience. However, their actual experience was the opposite. Several students meant that such concerns possibly did not relate to singular versus dyad placement, but on the surgery’s facilities and patient load. This was also the supervisors’ perception. One student said that because of their effectiveness working together, they saw more patients than other students did in singular placements.

I do not think I would have seen 14 patients in a day if I were alone. (Student 4)

### The student dyad was a safe space

Several dyad students had placement sites far from campus. They reported how their social life outside of the surgery influenced their positive experience during the placement period. One of the GPs also highlighted that dyad placement could be particularly suitable in rural places regarding spare time activities. All the students experienced the presence of another student as supportive in terms of social integration at the surgery. One student explained that having a fellow student who knew her made it easier to be herself from the start. Compared with previous clinical placements, she experienced better communication and faster integration with colleagues because she was in a dyad.

I think that by relaxing a bit from the start, it helps… I believe it removes a certain nervousness and edge that might cause one to overthink. (Student 4)

All the students expressed a feeling of safety in the presence of another student. One of the benefits of safety was that it led to open discussions with the supervisors. Students also felt more confident consulting with their supervisor after engaging in clinical reasoning with their peer. They encouraged each other to ask the supervisor for participation in activities that they considered enriching learning experiences. Several students explained that they supported each other in performing procedures.

I looked at my appointments, and there were things [procedures] I was not comfortable with. I told them, but then I knew that she [my peer] had done a lot more suturing at work (…) I wanted to learn, so I asked her if we could do it together (…) She did most of the work on the first patient, and I did most of the work on the next. (Student 5)

The students expressed that the large number of peer debriefs enabled by dyad placement was clearly beneficial. They had time and space to discuss patient encounters and situations directly after the consultations, during lunch and even after work. One student said that instead of having many questions, debriefing gave confirmation or correction, which was de-stressing. Another student highlighted that if an error occurred or if she said something that she regretted, debriefing with her fellow student, who was present and observing, decreased the feeling of shame.

I feel that if you don’t talk about it and never hear that others might feel the same way, it can become shameful and painful. But it was nice to ventilate a bit about those things. (Student 5)

### Students learned from each other’s clinical strengths

Several students stated that being observed during clinical work is not very common in medical school. Dyad placement made room for more observation performed by their peers. They appreciated focusing on talking with the patient while the observer could add questions at the end. Being an observer had educational benefits from performing clinical reasoning and considering possible treatment options. Several students said that they incorporated some of the elements their fellow student did well into their own consultations later. Observing the clinical examination also served as feedback for themselves and their own techniques.

It becomes a basis for comparison, academically, to see where you stand, right. Actually, it can be quite reassuring to observe a fellow student and think, yes, we’re about the same level, it’s going pretty well. (Student 2)

With diverse clinical experiences, the students expressed that they could be a resource for one another. Being able to help others helped them recognise their strengths, and they gained confidence. The students explained how they cooperated during patient advice, writing referrals and carrying out clinical procedures. In one dyad, one peer had experience in psychiatry and the other had experience in surgery.

I saw a child with question about autism. It was incredibly valuable to have her [the fellow student] in the room, and then she had a knee examination that I could assist with. (Student 1)

Students learned from discussing cases with their peers before the supervisor. Some drew parallels between conferring with peers in placement and discussing with colleagues in their future jobs as physicians. Through clinical discussions with peers, they felt ownership of the knowledge and remembered it. When discussing with a peer, both had to resonate towards the best solution.

It is perhaps the fact that none of us was the expert (…) that we involved ourselves more in the discussions in light of helping each other, compared to when one person knows and the other does not. (Student 5)

## Discussion

This study shows that dyad placements in general practice can provide students with opportunities to engage in peer teaching and peer learning. The support they provide to each other can enhance their learning processes. The supervisors experienced feasibility when having at least one consulting room and when there were sufficient patients available. Concerns regarding supervisor workload and less time to follow up each student were not experienced as challenging because of the students’ collaboration. Despite worries about not gaining enough clinical experience, the students did not perceive this as a problem in dyad placement.

There are many ways to make dyad placement work, and in our study the placements were explained as unique and tailored for the students forming the dyad. This heterogeneous practice reflects how it occurs in ‘real life’. General practice surgeries do not have the same prerequisites and structure to perform similar placements, and each meeting and relationship the students develop with the supervisors and staff is personalised and unique. Interestingly, all the students explicitly recommended their own way of organising the dyad placement. This is a challenge when the results from the study are to be transformed into departmental strategies and frames for future dyads. However, there may also be elements in addition to organisational aspects that can make a placement experienced successfully or not. An Australian study reported that students’ ability to participate in professional and social events such as conferences and receiving mentorship was associated with their satisfaction with decentralised education, including clinical placements [17]. A recent UK study exploring medical students’ experiences with rural primary care placements reported the importance of relationships with GP supervisors and other colleagues at the surgery. This, together with the adjustment to the rural environment, resulted in experiences like sense of belonging, autonomy, professional growth, and feeling valued [18]. When the students in our study recommended their placement site for dyad placement, they may have done this based on a total experience, including both organisational and relational elements.

Some concerns about dyad placement were refuted. The supervisors expressed initial concerns about the difficulty of following up each student properly. In another UK study, McPake et al. reported supervisors’ concerns regarding the assessment of a dyad when there were differences in students’ personalities, learning styles and capabilities, for example when one student was shyer than the other [19]. Nevertheless, the supervisors in our study explained how the flexibility within the student dyad to push, support, and help each other made the supervisor job less difficult regarding individualised follow-up. Also, the students’ initial concerns about gaining too little individual hands-on time during dyad training were refuted by experience. A study from Denmark reported medical students’ concerns after being in pairs in a course in history taking and clinical examination of real patients in their fourth year of medical education. The students expressed their appreciation of dyad practice in the initial stages of learning but were worried that continuous dyad training would decrease their clinical experience by giving them less time to practice with real patients [20]. In our study, the students concluded that this was not an issue, probably because of the effectiveness in dyads and possibilities for individual clinical work combined with collaboration.

The student dyad was a safe space, and the students reported safety as an important benefit of dyad placement. They explained in detail how being two students gave the feeling of safety which in turn led to actions such as asking to participate, daring to try and daring to speak up and ask questions. According to established learning theories, these behaviours can lead to learning, and several theorists have been inspired by John Dewey’s philosophy of learning seen as a process more than an outcome [21]. Amy Edmondson emphasised a concept (psychological safety) as a precondition for these learning behaviours in teams [15]. This concept roots back to clinical psychology in the 1950s and research on organisational change in the 1960s [22]. William Kahn defined psychological safety as ‘feeling able to show and employ one’s self without fear of negative consequences to self-image, status or career’ [23]. Edmondson reported that team psychological safety was associated with learning behaviour where team members seek feedback, share information, ask for help, talk about errors, and experimenting. These learning behaviours can, according to Edmondson, lead to outcomes such as adapting to change, greater understanding, or improved performance. From her team perspective, people must have mutual trust and respect for each other and be able to perform learning behaviours with a confidence that they will not be rejected, punished in any way or humiliated. The ‘safe space’ reported in our study represents the feeling of psychological safety generated within the dyad, which lowered the threshold for daring to ask to participate in learning situations and potentially take risks to show vulnerability or weaknesses. Notably, the supervisors play a pivotal role in facilitating thriving environments at the surgery, contributing to psychological safety [24, 25].

The students learned from each other’s clinical strengths by observing each other, helping each other and discussing clinical cases. This was also a finding from the Danish study from general practice [12]. These learning activities were reported by the students to enhance their self-reflection. A study exploring peer teachers’ learning from teaching highlighted that the teaching experience gave them insight into their own knowledge and also in its limitations [26]. Peer teaching is increasingly used in medical education for the practice of skills that students need in their future work as physicians [27, 28]. In our study, the students reported increased confidence and made parallels between student cooperation and working together as colleagues in a future clinical workplace.

### Strengths and limitations

We had 11 informants with their unique experiences, and we assessed the interviews as rich in terms of providing deep insight into the participants’ experiences. These insights have contributed to our understanding of future dyad schemes. During the research process, we evaluated the information power as satisfying according to the model of information power for qualitative studies [29]. The model emphasises the study aim, sample specificity, established theory, quality of dialogue and sample variation. Our study had a narrow aim, and the participants shared specific experiences from dyad placements. Theories were used to support interpretation of results in the discussion and not specifically in the analyses. We evaluated the dialogue quality as strong for most interviews, and since we also considered our sample relatively diverse, our assessment of information power was considered as satisfactory.

We found the use of a qualitative method to study experiences with dyad placement in general practice to be appropriate, given the study’s aim. Regarding educational benefits, we have not tested for outcomes but rather identified behaviours that theoretically and by evidence are related to learning. The study provides limited information on which pedagogical strategies the supervisors use in a dyad placement in general practice. Additionally, the study has explored less the experiences of differences in the supervision of two medical students compared to one at a time.

### Dyad placement in general practice: a second choice or an educational benefit?

Starting out as a second choice for student placement due to a lack of supervisors, this study has provided insights into positive experiences as well as potential limitations of dyad placements. The students’ stories about collaborating, resonating, debriefing and guiding each other are all within the concepts of peer teaching and collaborative learning. These concepts are well documented not to have any disadvantages in regard to learning outcomes [30]. In fact, peer teaching is recommended in educational programs for students’ professional development [31]. The safe space that the dyad created promoted an enriched learning environment according to the theory of psychological safety. Our study indicates that dyad placements in general practice can induce an educational benefit as well as decrease the number of necessary supervisors in a situation with increasing student numbers. Importantly, the collaboration between the students must not replace the facilitated reflection performed by the GPs. The presence of same-level peer teaching can function as an important supplement to the learning activities. However, discussing and reflecting upon core values of general practice is essential for learning about general practice as a discipline, and can be facilitated only by a physician in the field – the GP [32, 33].

### Implications and meaning of the study

This study revealed that there are various ways to run a successful dyad placement in general practice. This is an important message to supervisors that there is flexibility in tailoring placements according to local structures. We also learned that not all general practice surgeries have the best prerequisites for dyad placement. However, dyads can work as a good supplement to traditional one-to-one placement in general practice, not only when supervisor capacity challenges are present. GPs and universities should consider the benefits of dyad placement when planning clinical placements. Furthermore, sharing these results with medical students during their preparations for clinical placements could contribute to discussions and reflections on positive learning environments and how to be their own agents for learning in clinical placements. Both students and supervisors should be aware that their concerns about dyad placement will not necessarily play out in practice, as shown here.

Future research on dyad placements in general practice should include other stakeholders’ views, like administrative staff and patients. Exploration of suitable pedagogical strategies for dyad placement in general practice is needed, as well as investigating how dyad placement impacts doctor-patient dynamics.

## Conclusion

A dyad placement in general practice can be performed in several ways but requires adequate facilities and patient availability. Dyad placement promoted psychological safety for the students. They performed clinical reasoning in dialogue as well as peer teaching and learning of clinical skills. Despite the students’ and supervisors’ prior concerns, their experiences were positive and explained by professional collaboration and behaviour related to psychological safety. When dyad placement adds psychological safety and peer teaching, with their well-documented effects on learning activities, dyad placement in general practice can work as a good supplement to the traditional one-to-one placement structure.

## Data Availability

The datasets generated and analysed during the current study are not publicly available due to the consent obtained but are available (in Norwegian) from the corresponding author on reasonable request.

## Abbreviations

GPs: General practitioners
PAL: Peer-assisted learning
